# Evaluation of a Commercial Ballistocardiography Sensor for Sleep Apnea Screening and Sleep Monitoring

**DOI:** 10.3390/s19092133

**Published:** 2019-05-08

**Authors:** Dorien Huysmans, Pascal Borzée, Dries Testelmans, Bertien Buyse, Tim Willemen, Sabine Van Huffel, Carolina Varon

**Affiliations:** 1 KU Leuven, Department of Electrical Engineering (ESAT), STADIUS Center for Dynamical Systems, Signal Processing, and Data Analytics, 3001 Leuven, Belgium; sabine.vanhuffel@esat.kuleuven.be (S.V.H.); carolina.varon@esat.kuleuven.be (C.V.); 2 IMEC, 3001 Leuven, Belgium; 3 UZ Leuven, Department of Pneumology, 3000 Leuven, Belgium; pascal.borzee@uzleuven.be (P.B.); dries.testelmans@uzleuven.be (D.T.); bertien.buyse@uzleuven.be (B.B.); 4 Equilli, 2800 Mechelen, Belgium; tim.willemen@equilli.com

**Keywords:** ballistocardiography, pressure sensor, Emfit, home monitoring, sleep recording, sleep apnea, unsupervised learning, synchronization

## Abstract

There exists a technological momentum towards the development of unobtrusive, simple, and reliable systems for long-term sleep monitoring. An off-the-shelf commercial pressure sensor meeting these requirements is the Emfit QS. First, the potential for sleep apnea screening was investigated by revealing clusters of contaminated and clean segments. A relationship between the irregularity of the data and the sleep apnea severity class was observed, which was valuable for screening (sensitivity 0.72, specificity 0.70), although the linear relation was limited ($R^{2}$ of 0.16). Secondly, the study explored the suitability of this commercial sensor to be merged with gold standard polysomnography data for future sleep monitoring. As polysomnography (PSG) and Emfit signals originate from different types of sensor modalities, they cannot be regarded as strictly coupled. Therefore, an automated synchronization procedure based on artefact patterns was developed. Additionally, the optimal position of the Emfit for capturing respiratory and cardiac information similar to the PSG was identified, resulting in a position as close as possible to the thorax. The proposed approach demonstrated the potential for unobtrusive screening of sleep apnea patients at home. Furthermore, the synchronization framework enabled supervised analysis of the commercial Emfit sensor for future sleep monitoring, which can be extended to other multi-modal systems that record movements during sleep.

## 1. Introduction

Healthcare is evolving towards the application of automated systems for home-monitoring and pre-clinical screening to complement diagnostic routines. The current reference practice for diagnosis of sleep-related pathologies is a labor-intensive overnight stay in a specialized sleep center. There, a polysomnography (PSG) is performed, requiring the patient to wear encephalography electrodes, oronasal airflow sensors, thoracic and abdominal belts, electrocardiography (ECG) sensors, an oxygen saturation finger-clip sensor, a body position sensor, chin and leg electromyography and electrooculography sensors over a full night. This setup is highly obtrusive for the patient and impedes a normal night’s sleep. Moreover, the PSG procedure requires well-trained staff for analysis and is costly and burdensome. Sleep centers often have a limited capacity as well. Therefore, unobtrusive, cheap, and simple though reliable systems for monitoring at home are desired. These sensors could offer the ability to screen patients and prioritize them for hospital diagnostics, to increase healthcare accessibility or to enable long-term follow-up.

Among sleep disorders, obstructive sleep apnea (OSA) has the highest prevalence, from 13% to 33% in men and from 6% to 19% in women. However, these numbers are probably an underestimated and are likely to grow as they are closely associated with obesity and advancing age [1]. OSA is characterized by events of breathing disturbance causing hypoxaemia, large chest motions, and arousals from sleep. These events fragment the patient’s sleep and reduce phases of rapid eye movement and slow wave sleep. Consequently, OSA is an acknowledged risk factor for excessive daytime sleepiness, hypertension, and cardiovascular diseases [2]. The severity of sleep apnea is assessed by the Apnea–Hypopnea Index (AHI), which is the number of respiratory events (apneas and hypopneas) per hour. A patient is categorized as not suffering from sleep apnea (0≤ AHI <5), as having mild apnea (5≤ AHI <15), moderate apnea (15≤ AHI <30), or severe apnea (30≤ AHI) [3].

In order to expand unobtrusive resources for home-based sleep apnea screening and sleep monitoring, a commercial off-the-shelf sensor was explored, the Emfit QS (referred to as Emfit, developed and manufactured by Emfit, Finland). The Emfit is a pressure sensor built from electromechanical film (EMFi), which is a polypropylene film including gas voids. The material is similar to piezoelectric materials as a displacement charge is produced when a force is being applied. However, the change of the internal electric field is caused by the movement of static charges that were injected during fabrication of the film [4]. From the pressure-modulated signal, a respiratory signal and ballistocardiography (BCG) signal can be derived. The latter is an unobtrusive measurement of the body’s recoil caused by cardiovascular pulsation. As such, the sensor can provide information on sleep-disordered breathing as well as other origins of motion. A study by Koyama et al. [5], based on BCG, studied the feasibility of a piezoelectric sensor for apnea screening. They considered apneas during Cheyne–Stokes-like breathing to be correlated with AHI. This type of breathing is, however, only present in cardiac patients, thus targeting a subset of patients. Tenhunen et al. [6] evaluated a custom-made Emfit sheet and derived several parameters from breathing patterns to correlate these with AHI and assess sleep apnea severity. Despite the sensitivity of 0.95 in detecting subjects with AHI <15 using a combined parameter, the method required annotators to score breathing patterns visually and made no contribution to the automatic detection of these patterns. The same authors derived heart rate variability (HRV) as well [7], which resembled known HRV results of sleep apnea patients during periodic apneic events. This revealed an increase in sympathetic activity and claimed a good reliability of detection of periodic sleep disordered breathing. However, periods with wakefulness, movements, and artefacts were manually omitted, which hinders the application of Emfit as a stand-alone device.

**Figure 1:**
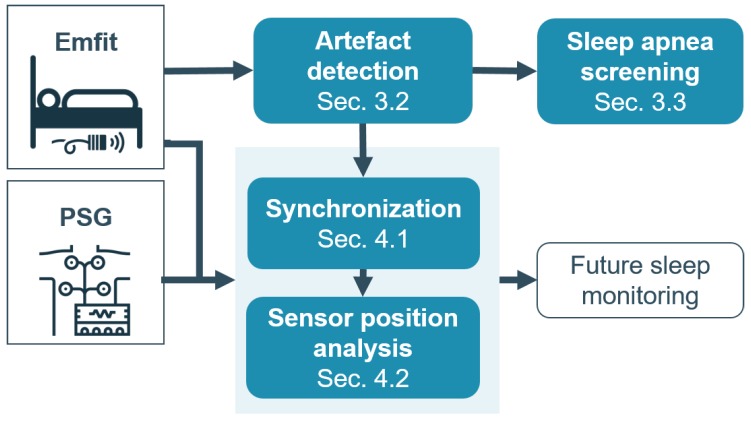
**Overview of study objectives.** First, the potential of the Emfit sensor for sleep apnea screening was investigated by searching for artefacts in the data caused by arousals and chest motions. Secondly, the study explored the suitability of this commercial sensor to be merged with gold standard polysomnography data for future sleep monitoring. Therefore, an automated synchronization procedure based on the previously detected artefact patterns was developed. After synchronization, the optimal position of the Emfit for capturing respiratory and cardiac information was identified.

Currently, no fully automated sleep apnea screening method has been established based on the Emfit sensor. Moreover, no Emfit studies have been performed using the commercial off-the-shelf Emfit sensor, according to the knowledge of the authors of this study. Hence, the goal of the present study was twofold (see Figure 1). First, the potential of the Emfit sensor in a stand-alone setting for sleep apnea screening was investigated. Sleep apnea is characterized by breathing cessations, which are terminated by arousals often accompanied by large motions of the chest. These arousals and chest motions cause deviations in the signals, which were referred to as artefacts. Hence, the Emfit data was explored to reveal clusters of artefacts and clean segments in the signal. The characteristics of these clusters were linked to the AHI. This cluster analysis was performed unsupervised as the Emfit sensor was not automatically synchronized with the PSG and to avoid burdensome manual labeling of the data into clean and artefact segments. Secondly, the study explored the suitability of this commercial sensor to be merged with gold standard polysomnography data for future sleep monitoring. Therefore, an automated synchronization procedure based on the previously detected artefact patterns was developed, since PSG and Emfit signals originate from different types of sensor modalities and cannot be regarded as strictly coupled. After synchronization, two different positions of the Emfit were investigated to find the optimal position for capturing respiratory and cardiac information similar to the PSG.

## 2. Materials

The Emfit QS is a commercially available pressure sensor (542 mm × 70 mm × 1.4 mm). Both the raw data and prefiltered data was made available. The raw data was sampled at 100 Hz. The prefiltered data contained a bandpass filtered signal at [0.08, 3] Hz and a bandpass filtered signal at [6, 16] Hz to obtain the respiratory and BCG signals, respectively. Filtering techniques were not specified by the manufacturer. From the PSG system (B3IP, Medatec, Belgium) the thoracic belt and ECG signal were analyzed.

**Figure 2:**
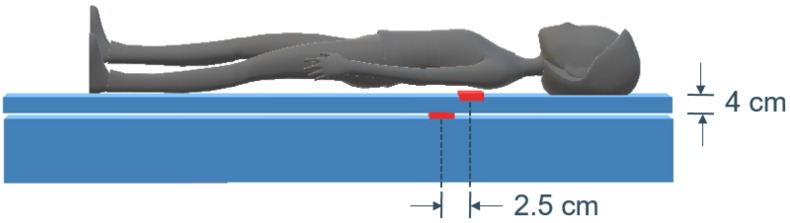
**Setup of Emfit sensors.** The bed consisted of a mattress with a mattress topper of approximately 4 cm thickness. One sensor was positioned underneath the thorax of the patient, separated by the mattress cover (position *Top*). A second sensor was placed beneath the topper (position *Bottom*) at a 2.5 cm horizontal distance to the top sensor.

In this study, two setups of the sensor were investigated. The bed consisted of a mattress on top of which a mattress topper of approximately 4 cm thickness was added. One sensor was positioned underneath the thorax of the patient, separated by the mattress cover (position *Top*). A second sensor was placed beneath the topper (position *Bottom*) at a 2.5 cm horizontal distance to the top sensor (see Figure 2). The horizontal distance ensured the limiting of the influence of the top sensor and compensated the effect of patients moving down in the bed when lifting the head of the mattress upwards. This setup was applied simultaneously in two beds in the sleep laboratory.

The Emfit sensor and PSG simultaneously recorded data for patients referred for sleep diagnosis in the sleep laboratory of the University Hospitals Leuven (UZ Leuven). Overnight PSG signals were annotated by sleep specialists according to the American Academy of Sleep Medicine. 2012 scoring rules [8] to derive the AHI. The dataset was recorded in two phases with an interruption of 7.5 months. The sensor setup remained the same; only the sensors were removed between phases and relocated as close as possible to the original location. Specifications of both datasets can be seen in Table 1. The last column, Top+Bottom, indicates the number of top sensor signals that have a corresponding bottom signal available. The reason for this was data loss due to technical problems, mostly with the bottom sensor.

All subjects gave their informed consent for inclusion before they participated in the study. The study was conducted in accordance with the Declaration of Helsinki, and the protocol with registration number B322201732928 was approved on November 8th 2018 by the UZ/KU Leuven Ethics Committee (Ethische Commissie Onderzoek UZ/KU Leuven).

## 3. Emfit-Based Sleep Apnea Screening

The Emfit sensor was evaluated in terms of its potential for sleep apnea screening in a stand-alone setting. As sleep apnea is characterized by breathing cessations that are often accompanied by large chest motions, these motions will induce deviations in the signal. These deviations will be referred to as artefacts, which, on the other hand, can also be induced by non-pathological body motions. It was hypothesized that the distortion of the data increased with AHI as more movement and arousals would be detected. Therefore, these artefacts were identified in the data by an unsupervised clustering method. First, the raw Emfit data was pre-processed. Thereafter, features were extracted that highlight irregularities in the signal. Features which optimally clustered the data were selected. Finally, the characteristics of the clustering were applied for sleep apnea screening.

### 3.1. Emfit Preprocessing

First, data quality was assessed by investigating the peak-to-peak amplitude (PP) distribution of the sensors after both measurement phases. Then, after subtraction of the mean value, the prefiltered respiratory signal of the Emfit sensor was further bandpass filtered to [0.08, 2] Hz. The respiratory signal was resampled at 4 Hz and the BCG signal at 50 Hz. As the signal amplitude was dependent on the weight and position of the patient, the signals were normalized. Normalization was based on the assumption that long-lasting periods of signal saturation corresponded to position changes by the patient. Segments between these periods were normalized by the median of the PP amplitude of this segment. If the median value was zero, the normalization of the previous segment was applied. This procedure was applied separately to the raw pressure, prefiltered respiratory, and prefiltered BCG signal. The periods of position changes and other saturated values were clipped to a value of 1, which was double the value of signals at the median amplitude.

Next, time–frequency domain information was extracted from the resulting signals by means of the discrete wavelet transform. To accentuate steep changes in the raw pressure signal indicating motion, a Daubechies 1 (i.e., db1 or Haar) wavelet was applied. Taking into account window size and sampling frequency, the signal was decomposed until level 8, i.e., [0.2, 0.4] Hz. The respiratory signal was approximated with a db4 wavelet (until level 3, [0.25, 0.5] Hz) and the BCG with db6 (until level 2, [6.25, 12.5] Hz). The respective wavelet shapes were chosen for their resemblance to the natural wave shape. A total of 16 signals (original signals and decompositions) were used for the subsequent feature extraction step.

### 3.2. Artefact Detection

#### 3.2.1. Feature Extraction

A feature window of 10 s was applied for sufficient time resolution and to include two to three breaths from the respiration signal. In total, 19 features were extracted in order to locate artefacts by inspecting outliers as well as irregularities (see Table 2). For features 9–19, the window was split into 3 equal subsegments over which PP was calculated, resulting in ${\mathrm{PP}}_{3}$ [9].

Time domain features were derived from both the untransformed signals and the three wavelet decomposed signals. These features were then normalized per subject using the z-score, and features with a Pearson correlation coefficient larger than 0.9 were removed. Lastly, feature values were transformed by means of the Euclidean norm normalization to decrease the effect of extreme values.

#### 3.2.2. Unsupervised Feature Selection

**Figure 3:**
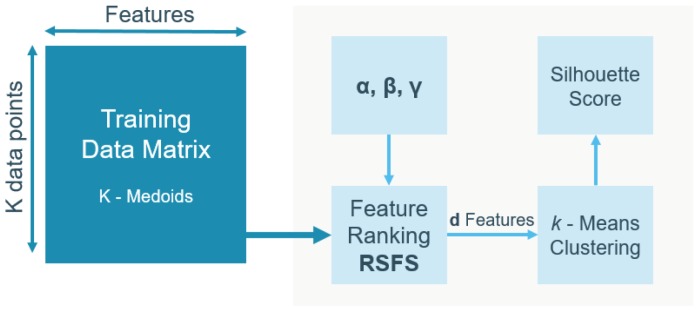
**Pipeline for unsupervised feature selection.** The input was a K-medoids clustering to reduce the dataset. This selection served as the input for unsupervised features selection. It was comprised of a parameter optimization that defined the feature ranking. The *d* top-ranked features were used for *k*-means clustering. The performance metric was the silhouette score. The pipeline was repeated for a different number of clusters *k*.

The unsupervised feature selection framework was based on Robust Spectral learning (RSFS) [10] (see Figure 3). This method provides a ranking of features, depending on three parameters of the RSFS objective function, i.e., α, β, and γ. Input feature vectors were taken from a reduced training dataset selected using K-medoids clustering with K=2000 and the Mahalanobis distance metric [11]. The K-medoids clustering was performed 100 times, such that the parameter optimization pipeline was run with 100 different training sets. Additionally, the Rényi entropy of every training set was calculated to verify the diversity within a training set and stability over training sets. Next, parameters α, β, and γ of the RSFS were taken from a 3D grid search over equispaced values in logarithmic scale from −3 to 3. For every set α, β, and γ, a feature ranking was calculated and a number *d* of top-ranked features was selected. Subsequently, a *k*-means clustering in a *d*-dimensional space was performed 20 times using squared Euclidean distance and random initialization. The clustering performance was evaluated by the overall average silhouette score [12]. The pipeline was iterated for d=[3,5,7] features and k=2 clusters. After completion of these iterative steps, the pipeline optimized the parameters α, β, and γ, resulting in the feature ranking, as well as the optimal number of features *d*.

#### 3.2.3. Clustering of Artefacts

With the optimized features, the training points were clustered using *k*-means with k=2. From this clustered training set, the centroids of both clusters were identified. These centroids acted as target points for the test data to determine its associated cluster by mapping every test data point to the closest centroid. The characteristics of the clusters were analyzed based on their feature values and a pairwise Mann–Whitney U test. As the features were tailored to detect large deviations in the signal, it was assumed that one cluster contained clean and the other contaminated, or artefact, data segments.

### 3.3. Screening of Sleep Apnea

Artefacts present in the Emfit signal originated from different sources such as position changes and apneic arousals. It was hypothesized that more severe sleep apnea patients would have more artefacts present in their data compared to healthier subjects. Clustering of these artefacts was performed using *k*-means clustering. This method assumes globular data structures due to the use of the Voronoi diagram. However, artefacted segments exhibited a varying morphology, resulting in less globular clusters. Therefore, some artefacted segments might be assigned to the clean cluster. The cleanness of the clean segment cluster was inspected by taking into account the distances of segments in the clean cluster to the clean cluster centroid. Outlying values were discarded by only considering values below the 95th percentile of distances. This segment distance distribution was calculated for every subject. A larger 95th percentile would indicate larger distances within the clean cluster and thus more artefact-like segments, hence a larger AHI was expected for the subject.

Training of the cluster centroids was performed with the dataset from Phase 1 (see Table 1). The dataset from Phase 2 was applied for testing by mapping the data of individual subjects to the trained centroids and evaluating the cleanness of the cluster based on the 95th percentile.

## 4. Emfit Integration with Polysomnography for Sleep Monitoring

### 4.1. Artefact Pattern-Based Synchronization

The Emfit is a stand-alone device that was not connected to the PSG. Therefore, both sensors were not automatically synchronized. A synchronization of the Emfit with the PSG is necessary for further analysis of the Emfit signal in a supervised manner. Synchronization based on timestamps of both sensors was not sufficient as large delays were still present. Also, simultaneously tapping the mattress with built-in sensors and marking the PSG data with a synchronization button was not sufficient as it was difficult to discriminate normal movement behaviour in the Emfit data during wake from tapping. Therefore, an automated synchronization procedure was developed based on the signals’ characteristics. To this end, the signal from the thoracic belt of the PSG was selected as a reference as its position was most proximate to the Emfit sensor. The Emfit respiratory signal and PSG respiratory effort signal, however, originate from different modalities. Therefore, a direct comparison of both signals based on clean waveforms was not possible as wave shapes can differ. The synchronization was based on the observation that movement of the patient and large changes in ventilation due to apneic arousal were reflected in both the Emfit as well as in the PSG. For this reason, the synchronization made use of the occurrence and pattern of artefacts in the signals, which were derived in Section 3.2.

#### 4.1.1. Polysomnography Preprocessing

The effect of movement caused by body posture changes was expected to be different in both modalities and more similar in the case of apneic breathing. Therefore, the central seven hours of sleep data were considered, as the patient was expected to be asleep. The PSG respiratory effort signal was bandpass filtered between [0.08–2] Hz using a Butterworth filter and downsampled from 500 Hz to 4 Hz. The data contained many small noisy peaks that were not necessarily present in both Emfit and PSG. To eliminate these, the top envelopes of the signals were derived using the secant method and a 1 s window.

#### 4.1.2. Delay Detection

**Figure 4:**
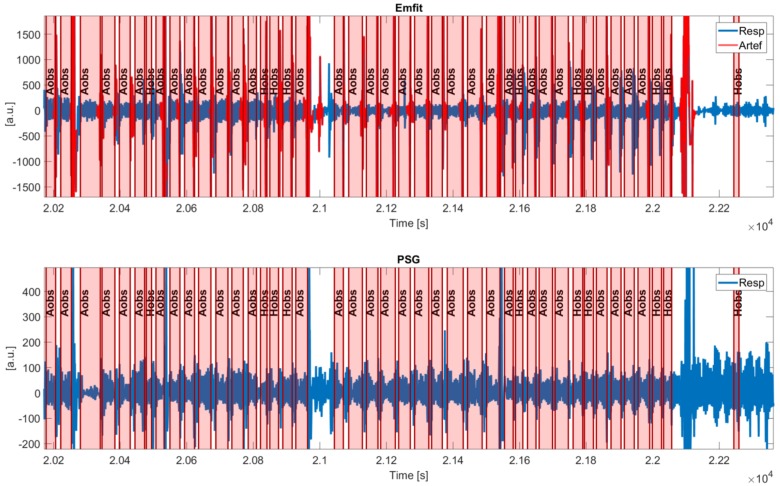
**Signal of patient with large AHI.** The signal contains consecutive apneic events, complicating synchronization.

**Figure 5:**
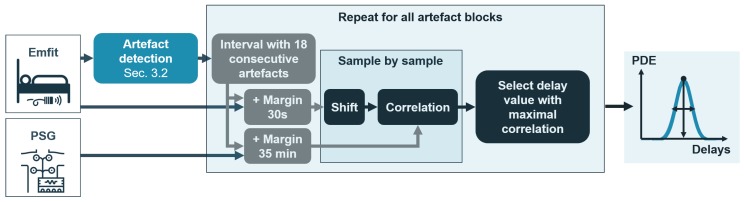
**Procedure for coarse delay detection.** The Emfit interval contained 18 artefact windows. The Emfit artefact block was shifted sample by sample along the PSG search interval. A probability density estimation (PDE) was derived over the series of optimal shifts.

The Emfit and PSG signals could have large delays as well as a large variation in delay between patients. Moreover, synchronization becomes more difficult if a very high number of distortions are present, often observed in patients with a very large AHI, as shown in Figure 4. Therefore, the synchronization was performed in two steps: a coarse delay detection and a refined delay detection. The coarse delay detection step took into account large artefact patterns, thus a signal interval containing 18 artefact windows (Section 3.2.3) was defined in the Emfit respiration signal. This interval was compared with intervals of the PSG signal by correlation (see Figure 5). A large margin (35 min) was taken, as the initial delay between signals could be substantial. The shift for which the maximal cross-correlation occurred was defined as the delay for the considered Emfit artefact interval. After iteration over all artefact intervals, the final delay value for the coarse synchronization was selected as the maximum of the probability density estimation (PDE) of delays. The bandwidth of the kernel indicated the standard deviation of the PDE and thus the certainty of the estimated delay. After shifting the signal with the coarse delay, it is necessary to consider more confined artefact blocks and precisely locate these in the PSG signal. The refined delay detection meant meaning a reduction of the interval to six artefacts and the margin to 5 min.

### 4.2. Sensor Position Comparison

**Figure 6:**
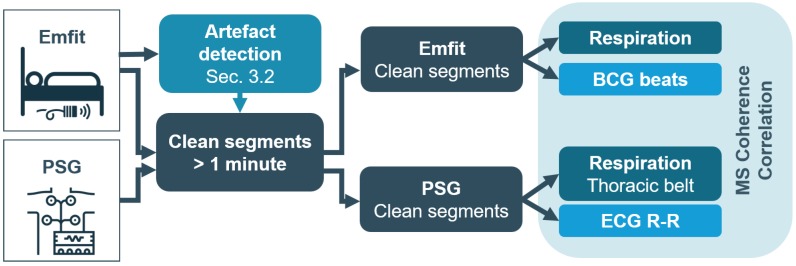
**Procedure for sensor position** **comparison.**

After synchronization of the Emfit with the PSG, the quality of the sensors was analyzed. The top sensor was expected to have a larger BCG signal quality, while the signal captured by the bottom sensor was attenuated by the mattress topper. The latter could lead to a better signal quality if many movement artefacts were present and for patients with an increased BMI. Without the attenuation, the signal would otherwise saturate. In a first phase, clean segments were extracted from the signals (see Figure 6). Based on the detected artefacts in the Emfit signal, segments of at least 1 min without an artefact were considered. These segments were compared to the corresponding segments from the PSG based on magnitude-squared (MS) coherence and correlation. Based on these statistics, the ability of the top and bottom sensors to capture heart rate and respiration information was assessed.

#### 4.2.1. Tachogram Derivation from ECG and BCG

The comparison between the BCG and ECG was based on heart rate information. Therefore, the tachograms of both signals and their evenly sampled interpolation was derived. First, the ECG signal was cleaned and saturated segments were not considered. Next, the R-peaks were detected using the algorithm proposed in [13]. Beats of the BCG signal were detected by an adapted Pan–Tompkins algorithm described in [14]. The tachograms of both sensors were analyzed for outliers by an adaptive threshold. It was defined as the running standard deviation of the 20 most recent samples multiplied by a factor of 5. Thereafter, the tachograms were interpolated and resampled to 4 Hz.

#### 4.2.2. Similarity Measures

Similarity was calculated between [0.1, 0.4] Hz for the respiratory signals of Emfit and PSG and between dynamic intervals for the interpolated tachograms of the BCG and ECG. For the latter, the maximum peak of the power spectral density of the ECG-derived tachogram in the low frequency (LF) band [0.03, 0.15] Hz and high frequency (HF) band [0.15, 0.4] Hz was determined. The frequency ranges covering the width at half maximum were considered. Additionally, the normalized cross-correlation was calculated between the HRV signals over lags in the interval [−15, 15] s.

In three cases, the clean segment was labeled as a segment containing no information, and no parameters were calculated: First, if the duration of one of the tachograms was smaller than 30 s; second, if the segment contained less than three detected heart beats; and finally, if the cross-correlation value was less than zero, as this indicates an erroneous tachogram of the BCG resulting from inferior data quality. The total length of clean segments over the total signal length was compared for the top and bottom sensors.

Since subjects have an unequal number of clean segments, some have a larger weight in the comparison as more of their segments are included. Therefore, a paired analysis was carried out as well. From every subject, the median values of the top and bottom parameter distribution were extracted and evaluated by a a Wilcoxon signed rank test. The complete parameter distributions for coherence and correlation were compared for individual subjects as well. It was evaluated whether the top or bottom performed significantly better and whether there was a relation with the BMI of the patients.

## 5. Results

### 5.1. Emfit Data Usability Assessment

Generally, the amplitude of the top sensors was higher compared to the bottom sensors.

The top sensors had similar median PP amplitudes in both beds during Phase 1 as well as during Phase 2. When comparing both phases, the top sensors of Phase 1 had a higher median PP amplitude compared to Phase 2. The manufacturer claimed to not have performed upgrades, which could have affected the recordings. Alteration in amplitudes could be explained by slight changes in location when reinserting the sensors between two phases.

A similarity in the amplitude of bottom sensors in both beds was also observed during Phase 1. However, in Phase 2 the distribution of median PP amplitudes of bottom sensor 1 was significantly different, with a median of only 21% compared to bottom sensor 2. Bottom sensor 1 might have shifted location during recordings in Phase 2 and was left out of the analysis.

### 5.2. Unsupervised Feature Selection and Clustering

**Figure 7:**
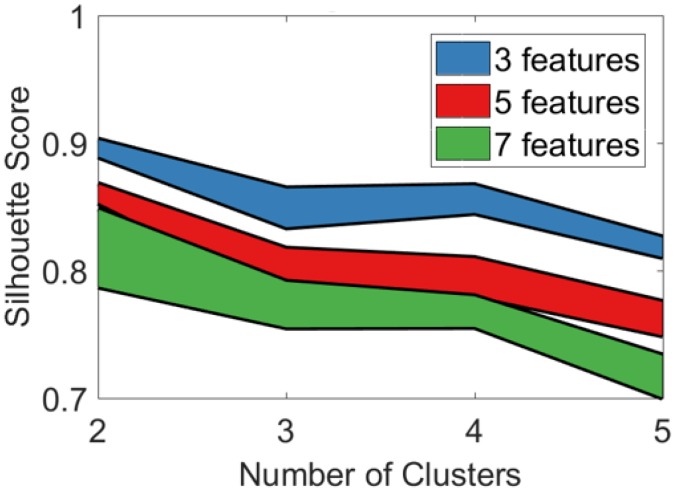
**Silhouette score distribution.** Borders indicate the 25th and 75th percentile of 100 iterations.

The pipeline was executed for d=[3,5,7] and a cluster number k=[2,3,4,5] and repeated for 100 different training sets. The resulting silhouette score distribution is displayed in Figure 7 (borders indicating the 25th and 75th percentiles). It can be seen that a limited number of features as well as a lower number of clusters resulted in higher silhouette scores. The decrease of the average silhouette score with a higher number of clusters k>2 suggested that the natural existing clusters might be split into multiple ones. Based on these results, the analysis was continued with feature number d=3 and cluster number k=2.

Optimal parameter sets {α, β, and γ} varied slightly, hence feature ranking and the resulting silhouette score varied as well over K-medoids iterations. Within 100 iterations, 2 optimal feature subsets were put forward, each with a 15% occurrence. The feature subset resulting in the highest average silhouette score was finally selected, being the features *pressure peakVar* at wavelet decomposition level 2, 3, and 4 (see Table 2).

Evaluation of Rényi entropy values (mean of 1.41, standard deviation of 0.040) indicated a limited variability (see Section 3.2.2). Therefore, a random training set was chosen and clustered with the optimized features. This resulted in an overall average silhouette score of both clusters of 0.91. Cluster 1 contained training samples with a highly varying silhouette score. In contrast, cluster 2 was a very well-defined cluster and should contain samples with similar characteristics. One cluster containing higher values of features, corresponding to higher peak variations, was labeled as *artefact* cluster. The other cluster characterized stable segments without intermittent peaks and was labeled as *clean* cluster. The difference in data distribution between clusters was high (Mann–Whitney U test, p<0.001), indicating that parameters were optimized to make a distinction between artefact and clean data. Mapping the test data to the trained centroids resulted in an overall average silhouette score for both clusters of 0.95.

**Figure 8:**
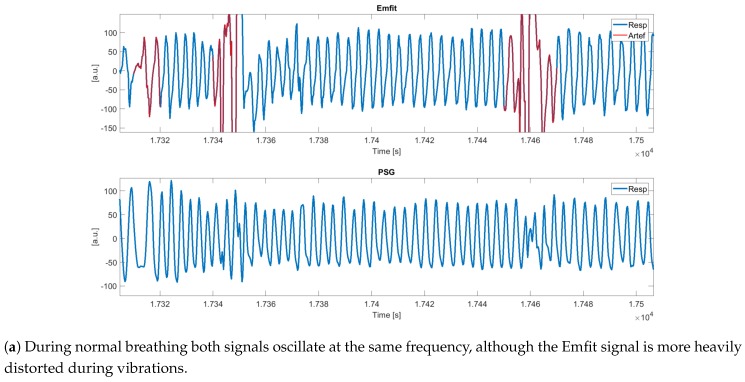

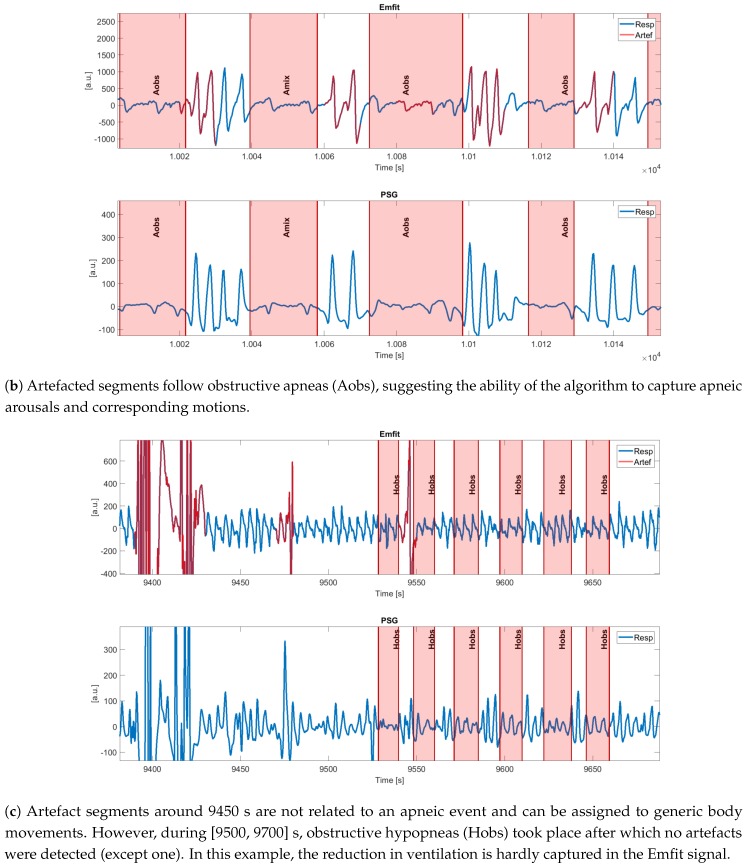
**Details of the synchronized Emfit and PSG signal with detected artefacts.** Segments are shown during normal breathing, obstructive apneas (Aobs), and obstructive hypopneas (Hobs).

Detailed examples of an Emfit signal with detected artefacts and (synchronized) PSG thoracic belt are displayed in Figure 8. Shaded intervals present apneic events and detected artefacts are indicated in red. Figure 8a illustrates that during normal breathing, both signals oscillate at the same frequency, although the Emfit signal is more heavily distorted during vibrations. Figure 8b shows artefacted segments following obstructive apneas (Aobs), suggesting the ability of the algorithm to capture apneic arousals and corresponding motions. Furthermore, Figure 8c displays artefact segments around 9450 s, which are not related to an apneic event and can be assigned to generic body movements. However, during [9500, 9700] s, obstructive hypopneas (Hobs) took place after which no artefacts were detected (except one). In this example, the reduction in ventilation is hardly captured in the Emfit signal.

### 5.3. Screening of Sleep Apnea

**Figure 9:**
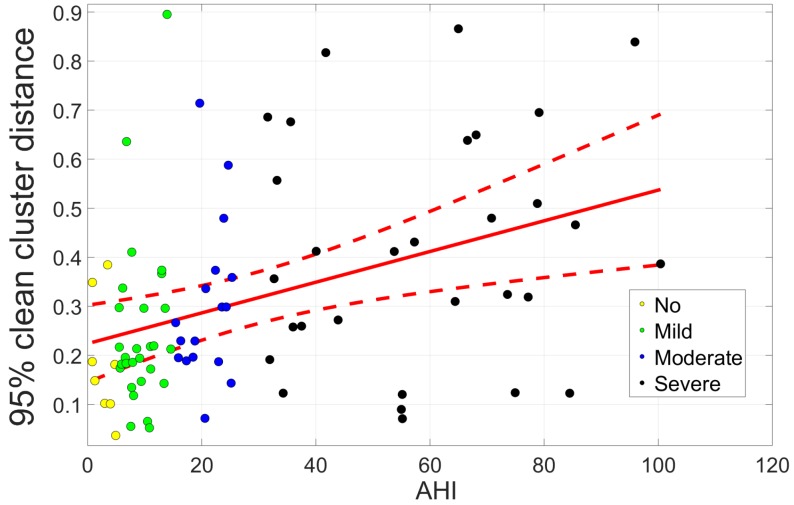
**Linear regression of the 95th percentile of distance to the clean cluster centroid with AHI.** The dashed lined is the 95% confidence interval with an $R^{2}$ value of 0.16.

As explained in Section 3.3, the cleanness of the clean segment cluster was inspected for every subject. For this, the 95th percentile of distance to the clean cluster centroid was derived. A linear regression of this metric with AHI is depicted in Figure 9. The regression displayed an upward trend; however, only a limited coefficient of determination $R^{2}$ of 0.16 was obtained.

**Figure 10:**
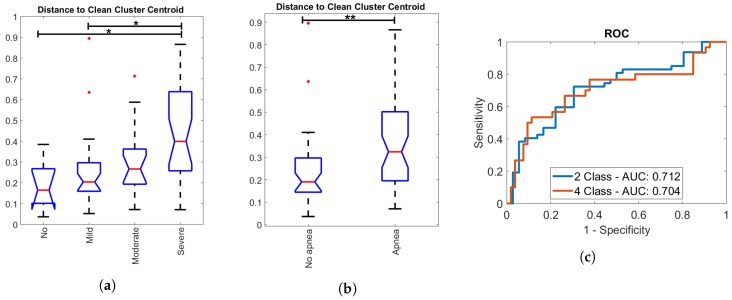
**Screening of sleep apnea patients.** The cleanness of the clean segment cluster was inspected for every subject by derivation of the 95th percentile of distance to the clean cluster centroid. These values were grouped according to the AHI of subjects. (**a**,**b**) A significant difference (Kruskal–Wallis test with Bonferroni correction, p<0.05) was established between no and mild apnea versus severe apnea, as well as between between patients with AHI <15 and 15≤ AHI (Mann–Whitney U test, p<0.05). (**c**) The ROC curves display the ability for screening of severe apnea patients (AHI ≥ 30) and more generally defined apnea patients (AHI ≥ 15).

The distance metric was also analyzed for standard sleep apnea classes of subjects, as shown in Figure 10, where the area under the curve (AUC) is defined of the receiver operating characteristic (ROC) curve. This also indicated a trend towards larger distances within the clean cluster and hence less regularity in the signal with increasing AHI. A Kruskal–Wallis test with Bonferroni correction between apnea classes indicated a significant difference (p<0.05) between no and mild apnea versus severe apnea. Furthermore, a significant difference (Mann–Whitney U test, p<0.05) was found between patients with AHI <15 and 15 ⩽ AHI. The ROC curve in Figure 10c displays the ability of screening of severe apnea patients (AHI ≥30), where a sensitivity of 0.77 and specificity of 0.62 was reached. The ROC curve for more generally defined apnea patients (AHI ≥ 15) reaches a sensitivity of 0.72 and specificity of 0.70. As a screening measure, a value of 0.229 for the 95th percentile of distance to the clean cluster centroid was taken.

Since the resulting feature set consisted of relatively simple and similar features (see Section 5.2), the screening performance was compared to a threshold-based method as well. After normalization of the data (see Section 3.1) and slicing into 10 s intervals, a window contained an artefact if any value exceeded the threshold. As such, the data of every patient was associated with a percentage of artefacts. Based on the artefact percentages and AHI of patients in the training data (Phase 1 of Table 1), an ROC analysis was performed. By analyzing the change in AUC with the selected signal amplitude threshold, an optimal threshold value was defined at 80% of the maximal amplitude. As such, a similar performance could be reached when screening patients from the test data (Phase 2). Here, the threshold was trained using the AHI labels. If the AHI is not available for training and an empirical threshold is taken at 50%, the results are close to random.

### 5.4. Artefact Pattern-Based Synchronization

The calculated delays of top and bottom Emfit sensors had a median value over all night recordings of 46.3 s ± 21.9 s. The accuracy of synchronization was verified by the bandwidth of the signal’s delay distribution. Fifty percent of the data had a bandwidth value below 3.68, 75% below 7.90 and upper adjacent of 14.26. Signals with a delay distribution bandwidth above 7.90 were visually checked. Empirically, bandwidths between 7.90 and 14.26 resulted in a synchronization error lower than or equal to 10 s. The error margin of 10 s was considered manageable as this can be compensated by a correlation based on ECG. This procedure, explained in Section 4.2.2, searches over an interval of [−15, 15] s for the highest correlation. Bandwidths above 14.26 exhibited a varying range of synchronization errors, which comprised 13.7% of the data. Six subjects had to be removed from further analysis as the actual delay after synchronization was still more than 15 s.

### 5.5. Sensor Positioning Comparison

**Figure 11:**
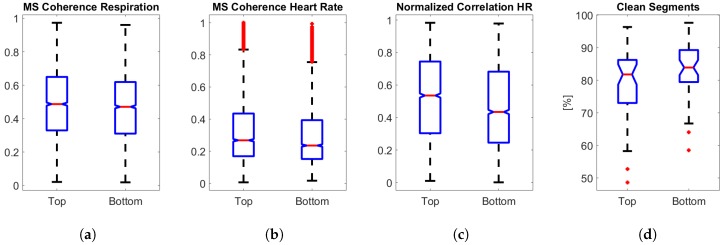
**Parameter comparison of top and bottom sensors over the whole population.** (**a**) Magnitude-squared coherence between Emfit and PSG respiration signals. (**b**) Magnitude-squared coherence between heart rate derived from BCG and ECG. (**c**) Cross-correlation between heart rate derived from BCG and ECG. (**d**) Percentage of clean segments that could be analyzed.

The parameters proposed in Section 4.2 were derived for all signals recorded by the top and bottom sensors (see Figure 11). Parameter distributions were similar for the top and bottom sensors; however, the median value of the top sensor was significantly higher. On an individual basis, in which the median value of distributions was taken into account, similar results were observed. The coherence parameters were significantly better for the top sensor, with p<0.05 and a correlation with p<0.001. On the other hand, the bottom sensor contained more clean segments (p<0.001). Concerning the influence of BMI on the optimal sensor position, no correlation could be found between these measures. Furthermore, the shift during ECG–BCG correlation analysis was taken into account. The median optimal shift over all signals was −0.15 s with a bandwidth of 0.25.

## 6. Discussion

The approach presented here demonstrated the potential for unobtrusive home-monitoring screening of patients at risk of sleep apnea with an off-the-shelf sensor intended for a home environment. Patients in which a large amount of artefacts are detected, due to position changes or apneic arousals, are considered as being at higher risk of suffering from sleep apnea. A trend was seen in the irregularity of the data with AHI (see Figure 10a), although the linear relation was limited ($R^{2}$ of 0.16). Moreover, a distinction was made between patients suffering from sleep apnea (15≤ AHI) and patients considered healthy (see Figure 10b). A significant difference existed between both classes, which is a beneficial result for screening purposes. Doctors are most interested in the identification of these patients as they should be referred for further research in a sleep clinic and ideally prioritized on the waiting lists. The screening with ROC analysis resulted in a sensitivity of 0.72, specificity of 0.70, and diagnostic odds ratio ($\mathrm{DOR}=\frac{\mathrm{sensitivity}\times \mathrm{specificity}}{\left(1-\mathrm{sensitivity}\right)\times \left(1-\mathrm{specificity}\right)}$) of 6.00. Investigation of misclassification revealed a trend in the BMI towards higher values for false negatives and false positives, which can be attributed to saturation of the Emfit pressure signal with heavy weight. As patients with $35\;\mathrm{kg}/m^{2}\leq$ BMI are known to have an increased risk for sleep apnea, these were removed from the screening analysis. This increased the DOR of the EMFIT screening method for 15 ⩽ AHI to 8.96. Additionally, different body positions can have an influence on the signal and resulting misclassification, such as lying higher, lower, or sideways.

A similar screening procedure was performed in [15], in which a larger sensitivity (80%) and specificity (87%) for severe sleep apnea screening were obtained. The study was based on the dataset of Phase 1 but using a leave-one-subject-out approach for testing. In the current study, a separate test set (Phase 2) was applied for screening. The sensors of the test set were slightly relocated compared to the training set. This relocation could have changed the properties of the artefacts and of the signal itself, thereby deteriorating the results. Therefore, pre-processing was improved by a normalization of the input data as well as the interpretation of the clustering results. A more gradual increase in irregularity of the data with AHI was observed in this study, complicating the screening of specifically severe sleep apnea patients (30≤ AHI).

In clinical practice, screening questionnaires for OSA are readily available. Chiu et al. [16] compared the screening performance of commonly used questionnaires such as the STOP-BANG questionnaire (SBQ), which was found to be a superior tool for detecting mild, moderate, and severe OSA. However, its sensitivity is high at the expense of low specificity (15≤ AHI: sensitivity of 0.90, specificity of 0.36, and DOR of 5.05), and its DOR is inferior compared to the current Emfit-based method. Nonetheless, taking into account the different ratios of sensitivity and specificity for both screening methods, these could be applied simultaneously to reinforce each other. Nevertheless, as a screening sensitivity of 0.95 and specificity of 0.92 based on manual annotation of Emfit signals was reached by Tenhunen et al. [6], improvement in automated methods is possible.

On this matter, clustering of data in clean and artefact segments was performed using *k*-means clustering, which is a method assuming globular data structures. However, artefacted segments exhibited a varying morphology resulting in less globular clusters, causing artefacted segments to be assigned to the clean cluster. A more complex clustering algorithm such as kernel spectral clustering [17] may be able to capture the varying morphologies of artefacts in multiple clusters. On the other hand, the simplified threshold method for screening performed similarly to the unsupervised clustering-based method. However, to establish an optimized threshold, the AHI of patients is required. In contrast, the clustering method is purely data-driven and is trainable without prior knowledge. Furthermore, its application can be extended to capture different types of irregularities in the data.

In order to establish an integration of the Emfit sensor with the PSG, an automated synchronization approach was developed. Segments in Figure 8 show that wave shapes in both modalities are different. As such, signals cannot be compared as a whole based on cross-correlations and the procedure focused on detecting large artefact patterns first with a coarse synchronization step. In patients with very a high AHI, synchronization becomes more difficult as signal deviations are almost continuously present.

The synchronization approach was automated by the introduction of a performance indicator, namely the bandwidth of the delay distribution. A threshold of a bandwidth = 14.26 could be defined to ensure sufficient synchronization accuracy. Moreover, most of the data (86.3%) attained a value below the threshold. However, some signals exhibited a delay distribution bandwidth above 15 while synchronization was accurate enough. A reason was that some patients leave the bed overnight. Electrodes are detached and only noise is recorded, causing the synchronization between both sensors to be distorted. The optimal shift before and after detachment is different, causing the bandwidth of the shift distribution to increase. Leaving the bed is a typical event, hence future work for Emfit–PSG integration should include the detection of electrode detachment and separate synchronization on different segments of the night. Concerning other recordings, the delay was fixed over the night. The difference in delay among recordings was suspected in instabilities during recording of the Emfit data, transmission over the hospital’s wifi network, or uploading to the Emfit server. Furthermore, synchronization in the signals of patients with a very large AHI (AHI > 90) was more difficult as artefacted segments were more similar due to almost continuous apneic events (see Figure 4). Different delays result in similar cross-correlation values. Additionally, signal quality tends to decrease, which causes the correlation value during synchronization to drop.

In a second stage, the sensor signals were precisely synchronized based on heart rate information instead of the respiratory signal. As the calculated delay between the tachograms of the ECG and BCG was small, a good synchronization was already reached during respiration-based synchronization. The presented framework for synchronization enabled a supervised analysis of the commercial Emfit sensor for future studies. Additionally, the framework can be applied to other multi-modal systems that record movements during sleep. This includes pressure-based signals of the thorax and respiratory-related signals, as simultaneous and similar artefacts can be expected in these signals.

Regarding the positioning of the Emfit sensor, it can be seen in Figure 11a–c that performance parameters exhibit similar distributions for the top and tottom. Parameters were only calculated for clean segments, therefore the percentage of (clean) segments included for analysis from every sensor was visualized in Figure 11d. From the bottom sensor, more clean segments of at least 1 min could be extracted as these signals were attenuated by the mattress topper and fewer artefacts were present in the signal. On the other hand, median values were significantly higher for the top sensor, indicating better sensor correspondence with the hospital’s PSG. This is due to the fact that the recorded signal amplitude of the bottom sensor was lower, making it more difficult for the algorithm to detect heart beats in the BCG. In general, MS coherence and correlation values of Emfit compared to PSG were modest. The Emfit sensor has a different measuring mechanism to the PSG thoracic belt or the PSG ECG. Therefore, different frequency components can be expected in Emfit respiration signals compared to the PSG thoracic belt. Moreover, the sensor quality of Emfit is expected to be less consistent during the night due to the different body positions of the patient.

## 7. Conclusions

A commercial pressure sensor was explored in terms of its potential for sleep apnea screening. An unsupervised algorithmic pipeline based on clustering was developed to characterize artefacts. A parameter based on the cleanness of these clusters was extracted as an indicator for sleep apnea severity. To enable a supervised analysis of the sensor for sleep monitoring, an automated synchronization procedure was developed based on the occurrence of artefacts in the respiratory signal. The synchronization framework can be applied to other multi-modal systems that record movements during sleep. This includes pressure-based signals of the thorax and respiratory-related signals, as simultaneous and similar artefacts can be expected in these signals. Furthermore, two different Emfit setups were analyzed for optimal signal quality. Locating the sensor as close as possible to the thorax and placing the sensor on top of the mattress is preferred if both respiratory and cardiac information are required. However, the positioning of the sensor is less critical if only respiratory information is required. Depending on the application, the signal-attenuating effect of a mattress topper could be advantageous.

## Figures and Tables

**Table 1:** **Datasets.** The dataset was recorded in two phases with an interruption of 7.5 months. The sensor setup remained the same; only the sensors were removed between phases and relocated as close as possible to the original location. The datasets are characterized by age, Body Mass Index (BMI), Apnea-Hypopnea Index (AHI), male (M) or female (F) and number of available signals from the sensors at the top and/or bottom location.

	#Patients	Age (years)	BMI ($\frac{\mathrm{kg}}{m^{2}}$)	AHI ($\frac{\mathrm{events}}{h}$)	M/F	#Top	#Bottom	#Top + Bottom
**Phase 1**	31	49.7 ± 11.7	31.3 ± 7.9	30.4 ± 25.7	27/4	31	22	22
**Phase 2**	83	46.8 ± 12.7	31.6 ± 6.2	28.9 ± 26.3	50/33	83	33	29

**Table 2:** **Features.** Nineteen features were extracted in 10 s windows.

	Feature
1–3	Mean, Variance (Var), Standard Deviation (Std),
4–5	Kurtosis, Skewness
6–7	Kurtosis of Autocorrelation, Shannon Entropy
8	Peak-to-Peak Amplitude (PP =max(x)−min(x))
	${\mathrm{PP}}_{3}$: individual PP of 3 equal subsegments of window
9	Maximum (${\mathrm{PP}}_{3}$) / mean (${\mathrm{PP}}_{3}$)
10–11	Var (${\mathrm{PP}}_{3}$) (peakVar), Std (${\mathrm{PP}}_{3}$)
12–16	[10%, 25%, 50%, 75%, 90%] (${\mathrm{PP}}_{3}$)
17	Interquartile Range (${\mathrm{PP}}_{3}$)
18	Interdecile Range (${\mathrm{PP}}_{3}$)
19	Median Absolute Deviation (${\mathrm{PP}}_{3}$)

## References

[1] Senaratna C.V., Perret J.L., Lodge C.J., Lowe A.J., Campbell B.E., Matheson M.C., Hamilton G.S., Dharmage S.C. (2017). Prevalence of obstructive sleep apnea in the general population: A systematic review. Sleep Med. Rev..

[2] Young T., Peppard P.E., Gottlieb D.J. (2002). Epidemiology of obstructive sleep apnea: A population health perspective. Am. J. Respir. Crit. Care Med..

[3] Kapur V.K., Auckley D.H., Chowdhuri S., Kuhlmann D.C., Mehra R., Harrod C.G. (2017). Clinical practice guideline for diagnostic testing for adult obstructive sleep apnea: An American Academy of Sleep Medicine clinical practice guideline. J. Clin. Sleep Med..

[4] Paajanen M., Lekkala J., Kirjavainen K. (2000). ElectroMechanical Film (EMFi)—A new multipurpose electret material. Sens. Actuators A Phys..

[5] Koyama T., Sato S., Kanbayashi T., Kondo H., Watanabe H., Nishino S., Shimizu T., Ito H., Ono K. (2015). Apnea during Cheyne-Stokes-like breathing detected by a piezoelectric sensor for screening of sleep disordered breathing. Sleep Biol. Rhythm..

[6] Tenhunen M., Elomaa E., Sistonen H., Rauhala E., Himanen S.L. (2013). Emfit movement sensor in evaluating nocturnal breathing. Respir. Physiol. Neurobiol..

[7] Tenhunen M., Hyttinen J., Lipponen J.A., Virkkala J., Kuusimäki S., Tarvainen M.P., Karjalainen P.A., Himanen S.L. (2015). Heart rate variability evaluation of Emfit sleep mattress breathing categories in NREM sleep. Clin. Neurophysiol..

[8] Berry R., Budhiraja R., Gottlieb D., Gozal D., Iber C., Kapur V., Marcus C., Mehra R., Parthasarathy S., Quan S. (2012). Rules for scoring respiratory events in sleep: Update of the 2007 AASM manual for the scoring of sleep and associated events. J. Clin. Sleep Med..

[9] Bruser C., Diesel J., Zink M.D.H., Winter S., Schauerte P., Leonhardt S. (2013). Automatic Detection of Atrial Fibrillation in Cardiac Vibration Signals. IEEE J. Biomed. Health Inform..

[10] Shi L., Du L., Shen Y.D. Robust Spectral Learning for Unsupervised Feature Selection. Proceedings of the 2014 IEEE International Conference on Data Mining.

[11] Varon C., Alzate C., Suykens J. (2015). Noise Level Estimation for Model Selection in Kernel PCA Denoising. IEEE Trans. Neural Netw. Learn. Syst..

[12] Rousseeuw P. (1987). Silhouettes: A Graphical Aid to the Interpretation and Validation of Cluster Analysis. J. Comput. Appl. Math..

[13] Varon C., Caicedo A., Testelmans D., Buyse B., Van Huffel S. (2015). A Novel Algorithm for the Automatic Detection of Sleep Apnea From Single-Lead ECG. IEEE Trans. Biomed. Eng..

[14] Willemen T. (2015). Biomechanics Based Analysis of Sleep. Ph.D. Thesis.

[15] Huysmans D., Buyse B., Testelmans D., Van Huffel S., Varon C. Unsupervised Artefact Detection and Screening Using Emfit Sensor in Patients with Sleep Apnea. Proceedings of the 45th Annual Computing in Cardiology Conference.

[16] Chiu H.Y., Chen P.Y., Chuang L.P., Chen N.H., Tu Y.K., Hsieh Y.J., Wang Y.C., Guilleminault C. (2017). Diagnostic accuracy of the Berlin questionnaire, STOP-BANG, STOP, and Epworth sleepiness scale in detecting obstructive sleep apnea: A bivariate meta-analysis. Sleep Med. Rev..

[17] Suykens J., Alzate C., Leuven K.U. Kernel Spectral Clustering: Model Representations, Sparsity and Out-of-Sample Extensions. Proceedings of the 4th International Conference on Computational Harmonic Analysis.

